# *Cecropia pachystachya* Leaves Present Potential to Be Used as New Ingredient for Antiaging Dermocosmetics

**DOI:** 10.1155/2019/8263934

**Published:** 2019-04-03

**Authors:** Maria Fernanda Fernandes, Jessica Leiras Mota Conegundes, Nícolas de Castro Campos Pinto, Luiz Gustavo de Oliveira, Jair Adriano Kopke de Aguiar, Elaine Maria Souza-Fagundes, Elita Scio

**Affiliations:** ^1^Laboratory of Bioactive Natural Products, Department of Biochemistry, Federal University of Juiz de Fora, 36036-900 Juiz de Fora, MG, Brazil; ^2^Glycoconjugate Analysis Laboratory, Department of Biochemistry, Biological Sciences Institute, Federal University of Juiz de Fora, 36036-900 Juiz de Fora, MG, Brazil; ^3^Department of Physiology and Biophysics, Biological Sciences Institute, Federal University of Minas Gerais, 31270-901 Belo Horizonte, MG, Brazil

## Abstract

Several biological activities have been reported for leaf extracts of* Cecropia pachystachya *species, including antioxidant and wound healing activities. This study aims to report, for the first time, the antiaging potential of the hydroethanolic (HE) and the ethanolic (EE) extracts obtained from the leaves of* C. pachystachya* using different* in vitro *assays. Both HE and EE presented relevant antioxidant capacity in different models, including phosphomolybdenum, 1,1-diphenyl-2-picryl-hydrazyl (DPPH), carotene/linoleic acid bleaching, and thiobarbituric acid reactive substances (TBARS) assays. Their ability to prevent the production of advanced glycation end products (AGEs) was also evaluated, and both extracts showed important activity, especially HE. The extracts also stimulated the fibroblasts proliferation* in vitro*, specialized cells that produce several mediators which maintain the skin integrity and youthfulness. Cytotoxicity of the extracts was not observed for this lineage or HEK-293, human embryonic kidney cells widely used to evaluate cytotoxicity of chemical compounds. HE also exhibited the ability to inhibit the collagenase (metalloproteinase MMP-2) and elastase activities. The total phenolic and flavonoids contents were also determined. HPLC analysis revealed the presence of the flavonoids orientin and iso-orientin, which were quantified to be used as chemical markers. The results suggested that the extracts of* C. pachystachya* leaves present the potential to be used in dermocosmetic formulations to prevent the skin aging process, which attracts the attention of pharmaceutical companies and researchers interested in the development of novel ingredients likely to be used as active principles in antiaging products.

## 1. Introduction

The normal functions and the natural appearance of the skin are severely affected by age and by many factors from the external environment which accelerate the skin aging, including ultraviolet radiation and pollution [1]. Nowadays, the search for new dermocosmetics capable of preventing and treating the skin aging process is gaining attention due to the increase in life expectancy and to the people concern in order to maintain a young appearance [2].

The harmful central mechanism that promotes skin aging is associated with the continuous solar ultraviolet radiation exposure which induces a complex and specific sequence of cellular responses, especially related to the synthesis of reactive oxygen species (ROS), which lead to noxious stimulus to the connective skin tissue [3]. Oxidative stress is also capable of altering the regulation of cellular mediators associated with the increase of metalloproteases expression, which are enzymes responsible for the degradation of extracellular matrix constituents, including collagen and elastin [4]. Also, ROS may promote direct oxidative damage in several cellular components, including organelle, nucleic acids, and plasmatic and mitochondrial membranes [5].

It is noteworthy mentioning that some histological changes are consequences of protein modifications secondary to oxidative reactions, including glycation reaction. For instance, dicarbonyl compounds produced by oxidative stress can bind to dermal matrix proteins, as collagen and elastin, which leads to the production of advanced glycation end products (AGEs). These chemical complexes may accumulate and accelerate the process of fibroblast apoptosis, aggravating the aging phenomenon [6, 7].

There is considerable interest in searching for new cosmetic ingredients that can be used as antiaging agents, notably those derived from natural sources [8]. In this context, the species* Cecropia pachystachya *Trécul have shown relevant potential to be used in many pharmaceutical formulations. The plant is popular known in Brazil as “embaúba”,“embaúva”,“imbaúba”,“umbaúba”, and “tore”, where the leaves are traditionally used to treat hypertension, diabetes, and pulmonary and cardiac disorders [9, 10]. Several pharmacological studies have reported the biological activities for different* C. pachystachya *leaves extracts. For instance, the methanolic extract showed significant hypotensive and anti-inflammatory activities [11, 12]. Besides, the ethyl acetate extract demonstrated strong wound healing effects [13]. It is worthy pointing out that both extracts showed significant antioxidant capacity [12, 13]. As the antiaging potential of natural substances is at least in part related to their antioxidant activity, those results are particularly impressive, as they encourage novel studies aimed to identify new extracts capable of reducing the aging process. Also, Duque et al. [13] reported that gel formulations containing ethyl acetate extract obtained from* C. pachystachya *crude extract increased the collagen deposition in rat skin wounds, which would be a relevant mode of action for a cosmetic principle.

Thus, the primary objective of this study was to assess the antiaging potential of* C. pachystachya* extract by evaluating its antioxidant, antiglycant, and its capacity to inhibit enzymes involved in skin aging like elastin and collagen. Ethanol and a mixture of ethanol-water were used as solvents as they are more feasible for dermal application in human skin, due to the lack of toxicity.

## 2. Materials and Methods

### 2.1. Reagents and Standards

Folin–Ciocalteu phenol reagent, 1,1-diphenyl-2-picrylhydrazyl (DPPH), 2,6-di-tert-butyl-4-methylphenol (BHT), *β*-carotene, linoleic acid, Tween 20, quercetin, orientin, iso-orientin, aminoguanidine, fructose, elastase, N-succinyl-Ala-Ala-Ala-p-nitroaniline, and collagenase were purchased from Sigma (St. Louis, MO, USA). Thiobarbituric acid (TBA) was purchased from Acros Organics (New Jersey, NJ, USA). All solvents used for HPLC analysis were HPLC grade from Tedia Company (Fairfield, OH, USA). Commercial chow was from Nuvital (Colombo, PR, Brazil). All other reagents were of the highest quality available.

### 2.2. Plant Material

*C. pachystachya *leaves were collected in Juiz de Fora, Minas Gerais state, Brazil, in August 2016. The plant was identified by a botanical specialist. A voucher specimen (CESJ 46591) was deposited at the Leopoldo Krieger Herbarium for future evidence (Federal University of Juiz de Fora).

### 2.3. Extract Preparation

Before the extraction process, the leaves were dried at 40°C and powdered using a knife mill (Marconi MA048, Piracicaba, SP, Brazil). The chemical constituents were extracted by maceration using two extractive solvents (ethanol 98% or ethanol: H_2_O 75:25) at a ratio of 1 g of powdered leaves to 10.5 ml of extractive solvent. For the ethanolic extract (EE), a maceration was accomplished for four days at room temperature, and then the solvent was evaporated using a rotatory evaporator (Tecnal, Araras, SP, Brazil) at 40°C. The hydroethanolic extract (HE) was obtained by heating the maceration system at 60°C for 30 min and then freeze-dried. Both extracts were kept in tightly stoppered bottles under refrigeration until the experimental procedures.

### 2.4. Total Phenolic Content

The total phenolic content was determined by the Folin–Ciocalteu method with some modifications [15]. This assay is based on a colorimetric oxidation/reduction reaction of phenolic compounds [16]. Both EE and HE were diluted in methanol in order to obtain a 500*μ*g/mL solution. Then, 120 *μ*L of Folin–Ciocalteu reagent, 100 *μ*L of calcium carbonate (4% m/v), and 30 *μ*L of the extract solutions were added in a 96-well plate. After 30 min of incubation time, the absorbance was measured at 770 nm using a spectrophotometer (Thermo Fisher Scientific, Waltham, MA, USA). The phenolic content was calculated using a calibration curve of tannic acid standard solutions (0.0075–0.2 mg/mL) and expressed as *μ*g of tannic acid equivalents (TAEs) per milligram of extract. All measurements were performed in triplicate.

### 2.5. Total Flavonoids Content

The amount of flavonoids was determined by aluminum chloride reagent assay, as described by Dowd [17], with slight modifications. Briefly, aliquots of 0.25 mg/ml of EE, HE, or reference standards were added to 0.25 ml of 2% aluminum chloride solubilized in a 0.5 ml of 5% acetic acid ethanolic solution. Then, 250 *μ*L was added to a 96-well plate (EE and HE at final concentration of 0.0625 *μ*g/mL). After 30 min of incubation time at room temperature, the absorbance was measured at 415 nm in a spectrophotometer (Thermo Fisher Scientific, Waltham, MA, USA). Water was used as blank control. Quercetin was used as reference standard for a calibration curve, which was constructed with seven points (1-320 *μ*g /mL). The total flavonoids content was determined in triplicate, and the results were expressed as the total amount of flavonoids (mg/g extract) in quercetin equivalents (QE).

### 2.6. HPLC Analyses

High-performance liquid chromatography was accomplished for orientin and iso-orientin quantification, using HPLC Agilent 1200 Series (Agilent Technologies, Santa Clara, CA, USA) at 25°C. An automatic injector and a Zorbax SB-C18 column 50 mm x 4.6 mm x 5 *μ*m were used. The mobile phase was consisted of a gradient elution of methanol: H_2_O, as described in Table 1. The flow rate used was 0.8 ml/min, and the detection was performed using a DAD 254 nm detector. The injection (20 *μ*l) was performed in triplicate.

### 2.7. Antioxidant Activity

#### 2.7.1. Phosphomolybdenum Method

The total antioxidant activity was evaluated by the phosphomolybdenum method according to Pierto et al. [18] with slight modification. Aliquots of 0.3 mL of EE and HE at 2 mg/mL was combined with 2 mL of a reagent solution (3 M sulfuric acid, 100 mM sodium phosphate, and 30mM ammonium molybdate). The absorbance was read at 695 nm after 90 min of incubation time at 95°C in a spectrophotometer (Thermo Fisher Scientific, Waltham, MA, USA). Ascorbic acid was used as the reference standard. The total antioxidant capacity was expressed as milligrams of HE or EE equivalent to 1 mg of ascorbic acid, using a seven-point standard calibration curve (50-300 mg/mL),

#### 2.7.2. DPPH Assay

The radical 1,1-diphenyl-2-picryl-hydrazyl (DPPH) was used for the determination of free radical-scavenging activity of both extracts [19]. Fifty microliters of HE or EE diluted in methanol at different concentrations (0.49–250 *μ*g/mL) were added to a 50 *μ*L of a methanolic DPPH solution (20 *μ*g/mL). After 30 min of incubation time, the absorbance was measured at 517 nm. The experiment was carried out in triplicate. Ascorbic acid was used as the reference standard. IC_50_ values were calculated using the software GraFit Version 5 (Erithacus Software, Horley, UK) and indicated the concentration of extract required to scavenge 50% of DPPH free radicals.

#### 2.7.3. Carotene/Linoleic Acid Bleaching Assay

Antioxidant activity was also determined by using the *β*-carotene bleaching assay according to Marco [20] with slight modifications. A stock solution of *β*-carotene-linoleic acid mixture was prepared as follows: 100 *μ*L*β* -carotene (10 mg/mL in dichloromethane HPLC grade), 30 *μ*L linoleic acid, and 265 *μ*L Tween 40 were dissolved in 500 *μ*L dichloromethane HPLC grade. Then, the dichloromethane was evaporated entirely using nitrogen gas. Also, 40 mL of oxygenated distilled water was added with vigorous shaking. Then, 250 *μ*L of this reaction mixture was dispersed to a 96-well plate. Finally, blank, HE, EE, or quercetin (74 *μ*g/mL), used as the reference standard, was added. The emulsion system was incubated for 2h at 45°C. The absorbance was read at 470 nm. The antioxidant activity (AA) was calculated regarding inhibition percentage relative to the control using

(1) $$\begin{array}{c} AA=\left[\;\frac{R\;\;control-R\;\;extract}{R\;\;control}\;\right]\times 100 \end{array}$$

R* control* and R* extract* mean rate blanching of control (quercetin) and extract, respectively.

#### 2.7.4. Thiobarbituric Acid Reactive Substances (TBARS) Assay

Oxidation products' synthesis was quantified using TBA reactive substances (TBARS) assay, as described by Sørensen and Jørgensen [21] with some modifications. Briefly, a mixture of 100 g of ground meat and 67 ml of distilled and deionized water with 7.5 mg/mL of each extract dissolved in methanol was thoroughly blended at room temperature until a smooth homogenate was formed. The mixture containing only meat, water, and methanol was used as the control. BHT 7.5 mg/mL dissolved in methanol was used as reference drug. Each mixture was transferred to amber jars and stored in a 5°C cold room over a period of 4 days. After this time, the percentage of meat oxidation was measured by the absorbance at 535 nm, using a spectrophotometer (Thermo Fisher Scientific, Waltham, MA, USA). The results were expressed in % oxidation inhibition. A calibration curve was prepared using malondialdehyde (MDA) standard, reacting with the TBA/phosphoric acid solution.

### 2.8. Antiglycant Activity

Antiglycant activity was* in vitro* determined using fructose-induced protein glycation models. The method was performed as described by Suzuki et al. [22] with some modifications proposed by Farsi et al. [23]. Stock solutions of fructose (1.6 M) and BSA (10 mg/mL) were prepared in 100 mM sodium phosphate monobasic monohydrate buffer (pH 7.4). Each solution was sterilized by vacuum filtration using Nalgene cellulose nitrate membrane filters (Fisher Scientific Ltd., Nepean, ON, CAN) before use. Incubation media containing BSA (10 mg/mL), fructose (1.6 M), sodium phosphate buffer (100mM), sodium azide (8g/L), and EE or HE at 12.5 *μ*g/mL) were prepared. Also, solutions containing aminoguanidine or quercetin, used as reference standards, or vehicle (75% EtOH: 25% H_2_O or 100% EtOH v/v) were prepared in 100 mM sodium phosphate.

After the incubation time at 37°C for 7 days, the amount of fluorescent AGEs formed was determined using a fluorimeter. The fluorescent intensity was measured at 330 nm (excitation) and 410 nm (emission). The antiglycant activity was expressed as percentage of fluorescent inhibition, using the vehicle as control:

(2) $$\begin{array}{c} AGE\left(\;\%\;\right)=\left[\;\frac{\left(F\;\;control-F\;\;sample\right)}{\left(F\;\;control\right)}\;\right]\times 100 \end{array}$$

F* control *and F* sample* mean fluorescence rate of control (vehicle) and extract or reference standards, respectively.

### 2.9. Enzymatic Activity

#### 2.9.1. Metalloproteinase-2 Inhibition

HE was incubated with matrix metalloproteinase-2 (MMP-2) obtained from RAW 264.7 supernatant culture for 10 minutes. Inhibition of MMP-2 was achieved by zymogram (10% acrylamide-bisacrylamide solution T 30% C 2.7% and gelatin 2 mg/ml) in Tris-glycine (25 mM/192 mM ) pH 8.3 containing sodium dodecyl sulphate (SDS, 0.1%). Incubation mixture (v/v) was mixed with equal amounts of 2× SDS-sample buffer containing 125 mMTris-HCl, 4% SDS, 20% glycerol, and 0.001% bromophenol blue in the absence of the nonreducing agent. After the migration, gels were washed with Triton X-100 (2%) and incubated with 50 mMTris-HCl, pH 8.2, containing 5 mM CaCl2 and 1*μ*M ZnCl2 and for 24h at 37°C. The gels were stained by Coomassie Brilliant Blue R-250 (0.5% dye, 30% methanol, and 10% acetic acid) and destained (30% methanol and 10% acetic acid) [14]. The activity of gelatinases was evidenced as bright regions (bleached) in the gel. The software Total Lab Quant® was used to measure the intensities of the bands.

#### 2.9.2. Elastase Inhibition

Elastase inhibition activity was determined according to the method of Bieth et al. [24] with some modifications. Two hundred microliters of 100 Mmtris-HCl buffer, 13*μ*L of 4,4 Mm N-succinyl-ala-ala-pro-val-4-nitroanilide (MAAPVN) and 20 *μ*L of HE were mixed and incubated for 15 min. Then, 5 *μ*L of 0.03 units/mL elastase (optimum reactivity of the enzyme) was added and incubated for another 15 min. The absorbance was read at 410 nm in a microplate. The inhibition rate was measured by the angular coefficient of the curves related to time x substrate concentration.

### 2.10. Cell Proliferation Assay

#### 2.10.1. Cell Lines

Cell line (BALBc/3T3) was acquired from the cell bank of Federal University of Rio de Janeiro. The cells were maintained in Dulbecco's modified Eagle's medium (DMEM) supplemented with the antibiotics penicillin (100 IU/mL) and streptomycin (100 *μ*g/mL), in addition to sterile fetal calf serum (FCS) 10% at 37°C under 5% CO_2_ atmosphere. The human embryonic kidney lineage, HEK293, was provided by Dr. Marcel Leist (University of Konstanz, Germany). Cells were cultured in DMEM medium (Dulbecco's Modified Eagle's medium) supplemented with 100 U/mL^−1^ penicillin and 100 mg/mL^−1^ streptomycin, and enriched with 2 mM of L-glutamine and 10% fetal bovine serum.

All lineages were incubated at 37°C in a humidified atmosphere containing 5% CO2 and split twice weekly. The cells were regularly examined regarding mycoplasma contamination and used until 20 passages.

#### 2.10.2. Cell Viability

Cell viability in the presence of HE and EE was determined using the MTT assay [25]. Briefly, BALBc/3T3 cells (1 x 10^4^ cells/well) were seeded in 96-well microplates and incubated for 24 h before the treatment. The cells were treated with various extracts concentrations (16.75; 32.5; 75; 150; and 300 *μ*g/mL) at 37°C, 5% CO2. Assays to evaluate cytotoxicity against HEK-293 cells were performed with the same cell density/well, but with a range of seven twofold concentration dilutions (1.56-100 *μ*g/mL). At the end of 44h of the incubation period, MTT solution (5 mg/mL in serum-free medium, 20 *μ*L/well) was added in each well and followed by incubation for 4 h. Finally, the MTT solution was removed, and the formazan crystals formed by MTT reduction were solubilized in 200 *μ*L of DMSO (BalBc/3T3 cells) or 200uL of 0.04M isopropanol. The absorbance was measured at 540 and 595 nm (respectively, to DMSO and isopropanol used to dissolve the formazan salt) using a microplate reader (Thermo Scientific, Waltham, MA, USA) and values were calculated in comparison to the control cells. At least, two experiments in triplicate were performed.

### 2.11. Statistical Analysis

The results obtained for each extract were analyzed statistically using one-way ANOVA followed by Tukey test for phosphomolybdenum, DPPH, carotene/linoleic acid bleaching, and enzymatic activity assays. Bonferroni test was used for lipid peroxidation and antiglycation activity. Results were presented as a mean± standard error. The GraphPad Prism 7.0 (GraphPad, San Diego, CA, USA) was used. Differences were considered statistically significant with* p*< 0.05.

## 3. Results and Discussion

### 3.1. Phenolic and Flavonoids Contents

Due to their antioxidant capacity, including the ability to chelate metallic ions and to scavenge free radicals, the phenolic compounds, especially flavonoids, are nowadays considered as indispensable components in a variety of nutraceutical, pharmaceutical, medicinal, and cosmetic products, including those destined to prevent the aging phenomenon [26]. Thus, for better positioning of any herbal drug intended to be used as the active principle of topical pharmaceutical formulations, it is interesting to verify its phenolic and flavonoid contents.

Although EE showed the highest total phenolic content, HE exhibited the highest total flavonoid content and higher yield (Table 2).

Flavonoids like orientin, iso-orientin, vitexin, isoquercetin, apigenin, and catechin were already identified in* C. pachystachya* leaves [27], which encouraged the HPLC analysis in the search for chemical markers.

### 3.2. HPLC Analysis

Although other studies have reported the identification of different flavonoids in* C. pachystachya* leaves, the HPLC analysis detected only the presence of orientin and iso-orientin. Probably, other flavonoids are present in quite small quantities in EE and HE, which may explain why they were not detected by the HPLC DAD detector. For this reason, only orientin and iso-orientin were used as chemical markers. Both were quantified in EE and HE, as shown in Table 3.

### 3.3. Antioxidant Activity

It is well known that the antioxidant activities of plant extracts are commonly related to the presence of phenolic compounds. Also, flavonoids are recognized as tissue protector against reactive oxygen species (ROS) [28]. The oxidative stress may lead to harmful cellular modifications, affecting the whole body, causing a variety of disorders, including diabetes, cancer, neurodegenerative diseases, and aging acceleration [26]. As a part of the natural aging process, endogenous defense mechanisms decrease, while the production of reactive oxygen species increases, resulting in an accelerated skin aging [29].

The free radical-induced aging theory, as proposed by Harman [30], suggests that aging and aging-associated degenerative diseases could be attributed to deleterious effects of ROS on various cell components [31]. Thus, it is intuitive to hypothesize that the topical application of antioxidant compounds may, at least in part, neutralize the skin aging process, and consequently decrease or prevent the external signs of skin aging [32]. Several methods have been employed for the assessment of antioxidant activities of plant extracts; however, the efficiency cannot be accurately evaluated by a single assay [33]. For this reason, four different methods were used to evaluate the antioxidant activity of EE and HE. The results showed that both extracts presented a significant antioxidant effect, as described in Table 4.

The IC_50_ values for EE and HE obtained by DPPH test were the same, and showed a rather high radical-scavenging activity, assuming that EE and HE are crude extracts endowed with several different chemical constituents at minor concentrations, unlike ascorbic acid, which is a pure compound. The TAC test [34], performed to evaluate the total antioxidant capacity of plant extracts, also reflected the strength of both extracts as antioxidants. The *β*-carotene/linoleic acid bleaching and TBARs methods were used to evaluate the ability of the extracts in preventing lipid peroxidation. The results clearly demonstrated that both extracts presented a relevant activity. EE was more effective in inhibiting MDA production, while HE presented more efficacy in the *β*-carotene/linoleic acid bleaching assay. Those results are quite relevant, as products of lipid peroxidation may react with cell macromolecules to form adducts with significant irreversible effects on cellular functions, which promotes the aging process [35].

### 3.4. Inhibitory Effect on BSA Glycation

In recent years, the anti-AGE potential of natural or synthetic compounds has gained particular attention by pharmaceutical companies interested in the development of novel antiaging products, as scientific studies have reported the substantial role of AGEs for the aging process [36].

AGEs are proteins to which a sugar molecule is bound, which can induce widespread tissue and cellular damage [37], as the original protein functions are inhibited. As skin contains several proteins, including collagen, the formation of these AGEs could be a viable explanation for the skin diminished functioning in aging [38]. Moreover, ROS and redox active transition metals accelerate AGEs formation [39].

The consequences of AGEs accumulation on the skin are a subject of interest in recent literature aiming at finding the causes for the old aspect of the skin and the decreased skin functions over the age. As in all organs, long-lived proteins are particularly prone to oxygenation. In the skin, this process affects mainly the dermal extracellular matrix proteins, including collagen, fibronectin, and elastin [40]. In addition, studies performed in synthetic skin revealed that the glycation process interferes with dermis metabolism, inducing to structural changes in elastic fibers, which reduce skin elasticity and increase MMPs activities [41]

In this study, the ability of EE and HE to inhibit AGEs formation was evaluated using the BSA-fructose assay, in which the bovine serum albumin is bound to fructose, leading to a glycated protein-sugar complex. The results showed that both EE and HE were capable of preventing the production of the protein-sugar complex. As described in Table 5, the extracts activities were expressive higher when compared to the reference standards (aminoguanidine and quercetin).

The antiglycation activity of the extracts, aligned to their antioxidant actions, reinforces the potential of HE and EE as possible active principle in pharmaceutical formulations intended to prevent skin aging.

### 3.5. Cell Viability

**Figure 1:**
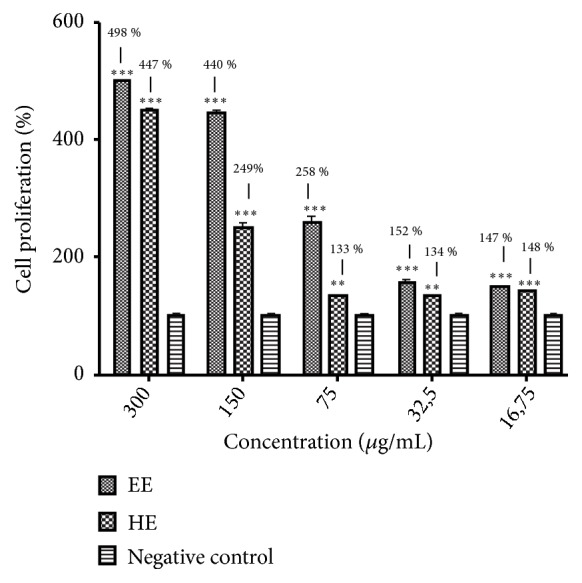
Percentage of BALB_C_/3T3 fibroblasts proliferation at different concentrations (16.15 to 300 *μ*g/mL) of EE (ethanolic extract) and HE (hydroethanolic extract) after a period of 24 h.

Dermal fibroblasts play critical functions at dermic homeostasis. Furthermore, fibroblasts can produce molecules which maintain skin integrity and youthfulness, so that substances capable of stimulating the fibroblasts proliferation may be used as ingredients in pharmaceutical formulations aimed to prevent cutaneous aging [42]. Thus, this study intended to evaluate whether the extracts are capable of inducing fibroblasts proliferation or cause any cytotoxic effects towards these cells. Cytotoxicity of the extracts was not observed for this lineage or for HEK-293 (data not shown), a human embryonic kidney cell that is widely used to evaluate the cytotoxicity of compounds [43]. EE and HE were investigated over a range of concentrations (16.75 to 300 *μ*g/mL). As shown in Figure 1, both extracts stimulated the fibroblast proliferation expressively, especially at high concentrations.

The results indicated that both extracts did not present cytotoxicity, at least at the concentrations tested, and stimulate notably the fibroblasts growth. These data corroborate to the study performed by Duque et al. [13], which reported that gels containing the ethyl acetate extract of* C. pachystachya* leaves stimulated fibroblasts proliferation and collagen deposition in rats.

### 3.6. Enzymatic Activity

The accumulation of free radicals in the skin is considered an inducing agent for collagenase, metalloproteinases, and elastase expression, accelerating dermal disruption [44]. Also, excessive exposure to sunlight, environmental pollution, and modern lifestyle habits, including smoking, accelerates the production of these skin enzymes, stimulating the degradation of the main components of the dermis extracellular matrix, as elastin and collagen [45].

Collagen is the most abundant protein in the extracellular matrix of the connective tissue of the human dermis [46]. This protein contributes to both skin strength and elasticity [47]. Elastase is a member of the chymotrypsin proteases family, which is primarily responsible for the breakdown of elastin, which is the main responsible for skin elasticity [47, 48]. Thus, compounds that can inhibit elastase and collagenase activities present potential to be used as cosmetic ingredients in pharmaceutical formulation developed to prevent skin aging, as they may reduce wrinkles formation [49].

In this context, plant extracts endowed with phenolic compounds have shown the ability to promote collagenase and elastase inhibition [44]. Flavonoids derived from plants have also the potential to bind metalloenzymes, due to their capacity to complex with metal ions [50]. Besides, Sim et al. [51] reported the structure-activity relationship of several flavonoids on collagenase (MMP-1) gene expression in UVA-irradiated human dermal fibroblasts and demonstrated that the inhibitory effect, at both the protein and mRNA level, became stronger with the increasing of OH groups on the B-ring of flavonoids.

Although EE and HE presented quite similar results in the chromatographic analysis, HE showed a total antioxidant capacity greater than EE. Besides, HE presented higher ability to inhibit AGEs production. The cell viability assay reported that at minor concentrations HE and EE showed very similar results. However, assuming that the use of low concentration of any chemical compound or natural product in pharmaceutical formulations is preferable to reduce adverse reactions and that HE showed higher TAC values and more potential to suppress AGEs production, the enzymatic assays were accomplished to evaluate the ability of HE to inhibit elastase and collagenase (matrix metalloproteinase-2, MMP-2) enzymes.

**Figure 2:**
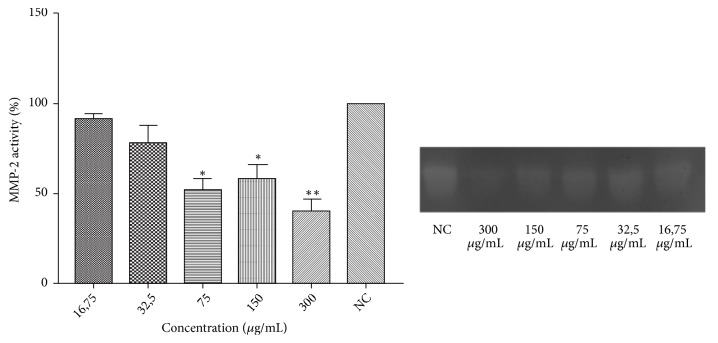
Inhibitory effects of hydroethanolic leaf extract (HE) on MMP-2 activity. A representative zymogram of MMP-2 activity obtained from RAW 264.7 is also shown. Electrophoresis in polyacrylamide gel at 7.5%, containing gelatin was performed. The metalloproteinase MMP-2 activities were quantified by bands densitometry and compared to the control [14]. Values expressed as mean ± standard error of the mean (n = 3). *∗* Statistically different from NC (*p*<0.05); ∗∗ statistically different from NC (*p*<0.01). NC: negative control (DMSO 0.05%).

**Figure 3:**
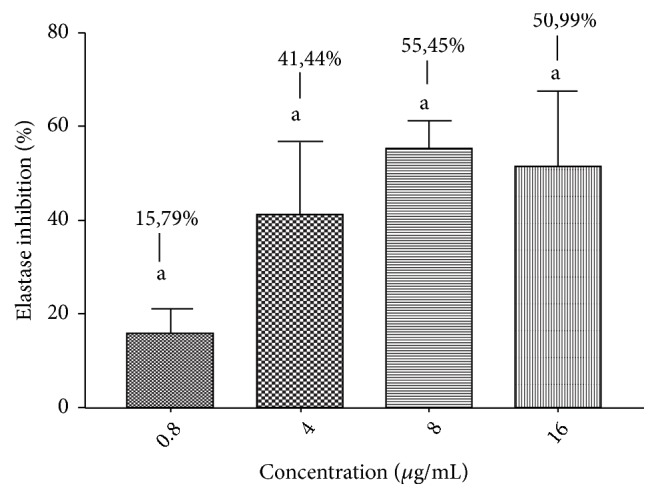
Inhibitory effects of hydroethanolic leaf extract (HE) on elastase activity. Gelatin zymography of MMP-2 was incubated for 24 h in the absence and presence of HE at different concentrations. Values expressed as mean ± standard error of the mean (n = 3). Equal letters represent no statistical difference (*p *< 0.05).

As the use of low concentrations of any active principle reduces the possibility of adverse reactions, it is reasonable to test the lowest possible concentrations in* in vitro* or* in vivo* preclinical assays. The lowest HE concentration that inhibited the elastase activity was 0.8 *μ*g/mL; however, the lowest concentration able to inhibit MMP-2 activity was 75 *μ*g/mL. As shown in Figure 2, HE reduced about 50% of the MMP-2 activity at 75, 150, and 300 *μ*g/mL. HE also inhibited elastase at all tested doses, as described in Figure 3; nevertheless, the activity notably increases at 4 *μ*g/mL, and it is only moderately higher at 8 and 16 *μ*g/mL.

## 4. Conclusion

The antiaging potential of* C. pachystachya *leaves is reported here for the first time. The results suggested that both ethanolic and hydroethanolic extracts are likely to be used in dermocosmetic formulations developed to prevent the skin aging process, as both extracts showed significant antioxidant and antiglycation activities. Those properties have been considered as an effective strategy to slow down human aging. Besides, the extracts were capable of inducing the proliferation of fibroblasts, which produce molecules that maintain the skin integrity and youthfulness. In addition, the hydroethanolic extract expressively reduced the activity of collagenase (MMP-2) and inhibited the elastase enzyme, which may be useful to prevent the extracellular matrix degeneration associated with the aging phenomenon.

## Figures and Tables

**Table 1:** Gradient elution used as the mobile phase for HPLC analysis.

Elution time (min)	Solvent		Flow rate
	Methanol	Water	

0	5%	95%	
5	33%	67%	0.8 mL/min
30	37%	63%	

**Table 2:** Yield and phenolic and flavonoids contents of the ethanolic (EE) and hydroethanolic (HE) extracts from *Cecropia pachystachya* leaves.

Extract	Yield	Phenolics	Flavonoids
	(%)^a^	(*μ*g ETA/mg DE)^b^	(*μ*g QE/mg DE)^c^

EE	10.37%	962.90 ±19.14	18.73± 0.12
HE	14.08%	656.16 ± 22.03	72.71± 0.92

^a^Data expressed as g of dry extract per 100 g of dried plant material.

^b^Total phenolic content was expressed as tannic acid equivalents (*μ*g of tannic acid per mg of dried extract).

^c^Total flavonoids content was expressed as quercetin equivalents (*μ*g quercetin equivalent/mg of extract powder).

**Table 3:** Amounts of orientin and iso-orientin in EE and HE.

Extract	Orientin^a^	Iso-orientin^b^
	(mg/g)	(mg/g)

EE	23.5 ± 0.5	31.8 ± 0.9
HE	35.5 ± 1.1	64.3 ± 1.6

^a^Line equation: y = 3611.2x – 47.859. R^2^ = 0.9946.

^b^Line equation: y = 2001.4x – 0.7367. R^2^ = 0.9997.

**Table 4:** Antioxidant activity of EE and HE by phosphomolybdenum, DPPH, *β* carotene/linoleic acid bleaching, and TBARs assays.

Sample	TAC	DPPH	*β*-Carotene/Linoleic Acid Bleaching Assay (%inhibition)	MDA (%inhibition)
	(EAA)	(IC_50_)		

EE	0.49 ± 0,07	^a^1.07 ± 0.05	^a^50.82± 0.65	^a^62.65±0.38
HE	0.53± 0,08	^a^1.07 ± 0.06	^b^65.18±5.82	^b^56.08±0.51
AA	-	^b^0.28 ± 0.05	-	-
Q	-	-	^b^71.28±3.42	-
BHT	-	-	-	^c^76.80±0.67

Values expressed as mean ± standard error (n = 3). Means with equal letters in the same column are statistically the same (p<0.05). AA: ascorbic acid; EE: ethanolic extract; EAA: equivalent to ascorbic acid; HE: hydroethanolic extract; MDA: malonaldehyde; TAC: total antioxidant capacity.

**Table 5:** Effect of EE and HE on BSA glycation at five different concentrations.

Extract or reference standard	Glycation Inhibition of BSA (%)
(12.5 *μ*g/mL)	

EE	^a^33.10% ± 0.17
HE	^b^51.30% ± 1.21
Quercetin	^c^8.01 % ± 1.26
Aminoguanidine	^d^23.79 % ± 2.68

Values are expressed as mean ± standard error of the mean (n = 3). Means with different letters in the same column shows statistical differences (*p*<0.001). BSA: bovine serum albumin; EE: ethanolic extract; HE: hydroethanolic extract.

## Data Availability

No data were used to support this study.
